# NR2F6 as a Disease Driver and Candidate Therapeutic Target in Experimental Cerebral Malaria

**DOI:** 10.3390/cells14151162

**Published:** 2025-07-28

**Authors:** Victoria E. Stefan, Victoria Klepsch, Nikolaus Thuille, Martina Steinlechner, Sebastian Peer, Kerstin Siegmund, Peter Lackner, Erich Schmutzhard, Karin Albrecht-Schgör, Gottfried Baier

**Affiliations:** 1 Research Program for Receptor Biochemistry and Tumor Metabolism, Department of Pediatrics, University Hospital of the Paracelsus Medical University Salzburg, 5020 Salzburg, Austria; v.stefan@salk.at; 2 Department of Biosciences and Medical Biology, University of Salzburg, 5020 Salzburg, Austria; 3 Institute for Cell Genetics, Medical University of Innsbruck, 6020 Innsbruck, Austria; nikolaus.thuille@i-med.ac.at (N.T.); martina.steinlechner@student.i-med.ac.at (M.S.); kerstin.siegmund@i-med.ac.at (K.S.); karin.albrechtschgoer@gmail.com (K.A.-S.); gottfried.baier@i-med.ac.at (G.B.); 4 Internal Medicine V (Hematology and Oncology), Medical University of Innsbruck, 6020 Innsbruck, Austria; sebastian.peer@i-med.ac.at; 5 Vienna Healthcare Group, Department of Neurology, Klinik Floridsdorf, 1010 Vienna, Austria; peter.lackner@gesundheitsverbund.at; 6 Karl-Landsteiner-Institute for Clinical Research in Acute Neurology, 1010 Vienna, Austria; 7 Department of Neurology, Medical University of Innsbruck, 6020 Innsbruck, Austria; erich.schmutzhard@i-med.ac.at

**Keywords:** experimental cerebral malaria (ECM), compromised blood–brain barrier (BBB) integrity, CD8^+^ T cell brain infiltration, nuclear receptor subfamily 2, group F, member 6 (NR2F6), innovative pharmacological therapy solution for CM

## Abstract

Cerebral malaria (CM) is the severe progression of an infection with *Plasmodium falciparum*, causing detrimental damage to brain tissue and is the most frequent cause of *Plasmodium falciparum* mortality. The critical role of brain-infiltrating CD8^+^ T cells in the pathophysiology of CM having been revealed, our investigation focuses on the role of NR2F6, an established immune checkpoint, as a candidate driver of CM pathology. We employed an experimental mouse model of CM based on *Plasmodium berghei* ANKA (*PbA*) infection to compare the relative susceptibility of *Nr2f6*-knock-out and wild-type C57BL6/N mice. As a remarkable result, *Nr2f6* deficiency confers a significant survival benefit. In terms of mechanism, we detected less severe endotheliopathy and, hence, less damage to the blood–brain barrier (BBB), accompanied by decreased sequestered parasites and less cytotoxic T-lymphocytes within the brain, manifesting in a better disease outcome. We present evidence that NR2F6 deficiency renders mice more resistant to experimental cerebral malaria (ECM), confirming a causal and non-redundant role for NR2F6 in the progression of ECM disease. Consequently, pharmacological inhibitors of the NR2F6 pathway could be of use to bolster BBB integrity and protect against CM.

## 1. Introduction

The parasite *Plasmodium falciparum* causes cerebral malaria (CM), which is a life-threatening complication of malaria. According to the WHO, CM is an encephalopathy characterized by an unarousable coma, primarily affecting children under five [1]. It is fatal in approximately 15–20% of cases. CM remains a significant health threat, accounting for around 95% of malaria-related deaths [2]. Furthermore, up to 25% of survivors experience long-term neurological problems, including seizures and neurocognitive deficits, which profoundly affect their quality of life [3]. CM patients are not currently offered any specific therapeutic interventions, other than anti-parasitic drugs and symptomatic treatment [4]. It is therefore of the utmost importance to further elucidate the pathophysiology and to identify druggable targets for therapeutic interventions.

The severity of CM has been largely determined by disruption of the blood–brain barrier (BBB) [5,6,7]. Increased permeability, which is caused by the breakdown of tight and adherens junctions between brain endothelial cells (ECs), comprises the barrier. This enables the entry of cytotoxic CD8^+^ T cells into the brain, which cause damage to neurons and glial cells through their inflammatory mediators and/or effector functions. Subsequently, intracerebral hemorrhage, increased intracranial pressure, and severe brain swelling can result in neurological dysfunction [8]. Mechanistically, and next to a dysfunctional BBB, recent research findings from mouse and human CM studies have provided causal evidence of CD8^+^ brain-infiltrating lymphocyte (BIL) sequestration as a key hallmark in the pathophysiology of CM [9,10,11,12,13].

Along this line of argumentation, our investigation focuses on the role of nuclear orphan receptor NR2F6, an established immune checkpoint of the CD8^+^ T cell effector compartment, as a candidate driver of cerebral malaria pathology. NR2F family members are known to regulate cell proliferation and differentiation in health and disease. NR2F6 has historically been identified as being involved in the development of the forebrain circadian clock and the early development of the *locus coeruleus* [14]. Within the immune system, NR2F6 inhibits the production of pro-inflammatory cytokines such as IL-2, IFN-γ, TNF-α, IL-17, IL-21, and IL-23 by interfering with the binding of NFAT, AP-1, and/or RORγt to critical promoter regions [15,16,17]. NR2F6 is regulated via PKC-mediated phosphorylation, which controls its release from DNA and consequently its ability to repress the transcription of the aforementioned cytokine genes [15]. As the loss of NR2F6 leads to hyperactive Th17 responses and heightened susceptibility to Th17-driven immunopathology [16,17], a protective anti-inflammatory role for NR2F6 in brain tissues during autoimmune encephalomyelitis has been established. Additionally, NR2F6 plays a pivotal role in regulating immune system activation during infection and oncogenesis. It accomplishes this by limiting the functions of T and NK cells, controlling cytokine production and maintaining immune system balance. Consequently, its absence enhances pathogen and tumor cell clearance by T cells and NK cells [15,18,19]. Overall, the multifaceted role of NR2F6 is to function as an intracellular immune checkpoint in adaptive and innate immunity, maintaining balanced immune activation to prevent excessive inflammation, which could otherwise lead to tissue damage or autoimmunity. Mechanistic research suggests that NR2F6 in T cells restrains antigen-specific effector responses, indicating that NR2F6 limits cytotoxic CD8^+^ T cell potential during infection. As these findings are also relevant to CM pathology, in which dysregulated immune responses and excessive pro-inflammatory cytokine production contribute to disease severity, this study aimed to determine the potential causal role of NR2F6 in experimental cerebral malaria (ECM) development and/or progression.

Remarkably, NR2F6 deficiency appears to mitigate rather than exacerbate pathology in ECM. This unexpected finding positions NR2F6 as a disease driver in this context. The enhanced survival benefit of *Nr2f6*-knock-out mice following *PbA* infection has been attributed to a less compromised integrity of the BBB. A reduced presence of cytotoxic T-lymphocytes within the brain, as well as less brain tissue destruction during ECM pathology, accompanied these effects. Therefore, inhibiting or modulating the NR2F6 pathway, an established regulator of both CD8^+^ T cells and intestinal barrier function [20], appears to be a unique and promising strategy to mitigate brain tissue damage, thus establishing NR2F6 as a potential therapeutic drug target for mitigating disease severity in CM pathology.

## 2. Materials and Methods

### 2.1. Animals

C57BL/6N and *Nr2f6*^−/−^ mice were housed and bred in the “Zentrale Versuchstieranlage” (ZVTA) in Innsbruck under specific pathogen-free (SPF) conditions. In this facility, the animals live at room temperature and with a 12 h light-dark cycle, while food (pellets) and water are provided ad libitum. As soon as pups can be weaned or until they reach the appropriate age for the *PbA* infection (6–8 weeks), the animals are transferred to the animal facility for infectious diseases, where they are housed in individually ventilated cages (IVC). All mice were backcrossed to a C57BL/6N background, as this strain is known to be susceptible to the development of experimental cerebral malaria. The experiments were in accordance with ethical and legal guidelines. The Austrian Federal Ministry of Education, Science and Research approved animal experiments at the Medical University of Innsbruck (BMFWF-66.011/0102-WF/V/3b/2017).

### 2.2. Parasites

ECM-causing *PbA* parasites, as well as the GFP-expressing *PbA* (MRA-867) that was used for the histological analysis, were kindly provided by Prof. Dr. Peter Lackner. The parasite was either cryopreserved in liquid nitrogen or continuously passaged from mouse to mouse. In this regard, mice that were older than 8 weeks and therefore less likely to develop cerebral malaria were used as transfer mice. Their blood was collected from the heart approximately on day 4 post-infection (p.i.) in 500 I.U. heparin and was transferred to the next mouse.

### 2.3. Infection of Mice with PbA

Mice were anesthetized with isoflurane and injected with 50 µL of a blood/NaCl (0.9%) mixture containing 7.5 × 10^4^ iRBCs (infected red blood cells) intravenously (i.v.) for infection in any experiment. The *PbA*-infected erythrocytes were obtained by collecting heart blood in heparin (500 I.U., Gilvasan, Vienna, Austria) of a previously infected transfer mouse. This was performed once the passage mouse had reached a stage of parasitemia, where all forms of the parasite in its blood stage are represented (approximately day 4 p.i.). Based on the count of erythrocytes in the blood (assessed with DeNovix CellDrop fluorescent cell counter) and the parasitemia, as determined by blood smear, the 7.5 × 10^4^ iRBC injection solution was prepared.

### 2.4. Evaluation of Disease Progression

To assess disease progression, the mice were evaluated every day using the Innsbruck Cerebral Malaria Score (ICMS). This score is an adaption by Lackner et al. [21] of the SHIRPA score by Rogers and colleagues [22]. It is designed to detect neurological deficits that develop during ECM. The ICMS includes ten neurobehavioral tests, evaluating body position, spontaneous activity, piloerection, grip strength, gait, straddle response, touch escape, irritability, vocalization, and body/abdominal/limb tone. Additionally, body temperature, weight, and parasitemia were assessed. In case a mouse achieved less than 8 out of 18 points in the ICMS, lost more than 20% of body weight, manifested a body temperature below 35 °C, or showed a parasitemia above 40% iRBC, the mice were euthanized immediately.

### 2.5. Parasitemia

To assess the amount of *PbA*-infected red blood cells (parasitemia), blood from the tail tip was collected on a glass slide and used to generate a thin blood smear. The samples were fixed for 7 min in methanol (≥99.5%, Fisher Scientific, Pittsburgh, PA, USA), stained for 2 min with Giemsa (Sigma-Aldrich, St. Louis, MI, USA), and subsequently rinsed with deionized water. Parasitemia was calculated according to the following equation:

$$\frac{\mathrm{number}\;\mathrm{of}\;\mathrm{iRBC}}{\mathrm{number}\;\mathrm{of}\;\mathrm{total}\;\mathrm{erythrocytes}}=\mathrm{parasitemi}$$

### 2.6. Assessment of Blood–Brain Barrier Integrity

In order to evaluate the integrity of the BBB, an Evans Blue (EB) permeability test and a brain-swelling assay were performed as previously described [23]. In brief, on day seven after infection with *PbA*, 100 µL of 2% Evans Blue/PBS (*w*/*v*) was injected i.v. into age-matched (6–8-week-old) female *Nr2f6*^−/−^ and C57BL/6N WT mice. At 1.5 h post-injection, the mice were terminally anesthetized with 100–150 µL of ketamine (100 mg/mL)/xylazine (20 mg/mL) (Livisto)/NaCl (1:1:2) (i.p.) and perfused through the left ventricle with 0.9% NaCl (Fresenius Kabi) for 5 min merely driven by gravitation. Subsequently, the brains were isolated, and the two hemispheres were separated. One hemisphere was weighed and dried at 150 °C for seven hours. Thereafter, the brains were weighed again to assess the amount of drying loss. Simultaneously, the other half was used to determine the concentration of Evans Blue in the brains to evaluate the integrity of the BBB. Therefore, the brains were transferred to 15 mL centrifugation tubes containing 2 mL formamide (2%) and shredded with a tissue rupture (Qiagen, Venlo, the Netherlands). The suspensions were incubated at 37 °C in the dark for 48 h. An Evans Blue standard curve was prepared, and the samples were centrifuged for 15 min at 14,000 rpm. A total of 300 µL of each sample and standard was transferred to a 96-well flat-bottom plate, and the absorption was measured at 620 nm.

### 2.7. Isolation of Brain-Sequestered Lymphocytes

As described above, brains from *PbA*-infected mice were excised on day 6 or 7 post-infection. To isolate brain-infiltrating lymphocytes for FACS experiments, brains of WT and *Nr2f6*-knock-out mice were transferred into 1 mL digestion mix (2.5 mg/mL Collagenase A (Sigma-Aldrich, St. Louis, MI, USA), 1 mg/mL DNase (Sigma-Aldrich), 2.5 mM MgCl, and 2% FCS in 1 × PBS) and cut into small pieces. After storing this mixture for 30 min at 37 °C, 0.01 M EDTA (ThermoFisher, Waltham, MA, USA) was added and incubated for another 5 min at 37 °C. Thereafter, brains were pushed through a 100 µm cell strainer and rinsed with 7 mL RPMI+++ (RPMI (PAN Biotech) + 1:100 penicillin/streptavidin, 10% FCS, 1:100 L-glutamine). A Percoll (GE Healthcare) density gradient centrifugation was performed to isolate the lymphocytes (30%, 70%). The samples were centrifuged at 500× *g* at RT for 30 min (acceleration 5, no brake). A total of 3 mL of the interphase with lymphocytes was collected. The lymphocytes were transferred into 10 mL of fresh cRPMI and centrifuged again at 300× *g* at RT for 7 min. These cells were then used for flow cytometry.

### 2.8. Flow Cytometry

For flow cytometric analysis of lymphocytes in the brain, mice were anesthetized as described above. The brain was harvested after perfusion for 5 min with 0.9% NaCl, and lymphocytes were isolated from the brain as described above. After centrifugation at 300× *g* for 7 min at 4 °C, erylysis was performed for 3 min at RT (2 mL of 1x erythrocyte-lysis buffer (145.6 mM NH_4_Cl, 0.127 mM EDTA, 11.9 mM NaHCO_3_ in ddH_2_O, pH = 7.3)). The suspension was filtered through a 40 µm cell strainer, which was rinsed with 8 mL RPMI. Cell pellets were resuspended in 200 µL cRPMI (complete RPMI+++, 1:100 non-essential amino acids (Sigma-Aldrich), 1:100 sodium-pyruvate (Sigma-Aldrich)) and transferred to a 96-well flat-bottom plate, which has been coated with 5 µg/mL anti-CD3, for overnight stimulation. Subsequently, the cells were stained with a fixable viability dye eFluor 780 and the respective antibodies (Table 1) while they were fixed and permeabilized (wash/perm, BD Biosciences, Franklin Lakes, NJ, USA) for intracellular staining. Samples were analyzed using a BD FACS CantoII^TM^.

### 2.9. Histology of Brain Sections

Histological investigation of the brains was performed using Hematoxylin–Eosin (H&E) staining to assess brain lesions. The parasites and the CD8^+^ T cells sequestered in the brain microvasculature were visualized by immunofluorescence staining. Therefore, mice that were infected with GFP-*PbA* were terminally anesthetized on day 6 or 7 using 100–150 µL of a ketamine (100 mg/mL)/xylazine (20 mg/mL)/NaCl (1:1:2) mixture (i.p.). They were perfused for 5 min with 0.9% NaCl (Fresenius Kabi) through the left ventricle. Subsequently, brain tissue was fixed with another 5 min of perfusion using 4% Paraformaldehyde (PFA). The brains were excised and kept in 4% PFA overnight at 4 °C. The cerebral tissues were transferred to a sucrose solution (Sigma) for gentle freezing the following day. For IHC, fixed brains were cut sagittally in 20 µm slices with a cryotome, and the slices were dried at RT for four hours. Subsequently, brain sections were blocked for 1 h at RT using 2% NGS (normal goat serum), 1% BSA, and 0.3% Triton-X100 in PBS (Sigma-Aldrich). Primary antibodies against CD8 cytotoxic T cells (rabbit, 1:500) and CD31 as an endothelial marker (rat, 1:50) were prepared in 0.5% BSA and 0.3% Triton-X100 in PBS and incubated for 1 h at 4 °C. Afterwards, three wash steps, each 5 min, using PBS were conducted. The secondary antibodies, goat-anti-rabbit (Alexa Fluor 647) and goat-anti-rat (Alexa Fluor 568), were prepared at 1:1000 in 0.5% BSA and 0.3% Triton-X100 in PBS and incubated for 1.5 h at room temperature. Slides were washed three times in PBS for 5 min before adding DAPI-containing ProLong Diamond Antifade Mountant (Invitrogen, Waltham, MA, USA) and cover slides. The GFP-*PbA* parasite could be visualized by its GFP labelling. Images were acquired using the LSM 700 Axio Observer.Z1m confocal microscope with the AxioCamMRm camera and Zen LSM software (Zeiss, Oberkochen, Germany, version 2.0), which was also used for further image processing. For H&E staining, brain sections were incubated for 90 s in Hematoxylin/Hemalum (Roth) and subsequently washed in tap water for 15 min. Furthermore, the sections were stained with Eosin Y solution by Roth (+1 drop of anhydrous acetic acid per 100 mL) for 10 s. Brain tissues were dehydrated in an ascending ethanol series and fixed with Histoclear. Finally, sections were imaged with a light microscope and 10× objective (Olympus BX43, camera Progres CT3, Gryphax Arktur). Lesions were quantified, and their size was assessed with ImageJ (version 1.54).

### 2.10. Statistical Analysis

Statistical analysis was performed using GraphPad Prism (San Diego, CA, USA, version 9.0), which was also used for the creation of graphs. Results are depicted as arithmetic means ± standard error of the mean (SEM). In order to assess the statistical significance, two-tailed, unpaired Student’s *t*-tests were performed. Yielding a *p*-value < 0.05, the difference was considered significant (*), while highly significant results with a *p*-value < 0.01 or even <0.001 were marked with “**” or “***”, respectively. In case a mouse manifested an unusually low parasitemia that was not in accordance with a typical disease progression, it was excluded from the analysis.

## 3. Results

*Nr2f6*-knock-out mice showed attenuated disease progression and a significant survival benefit over wild-type (WT) mice in ECM.

**Figure 1:**
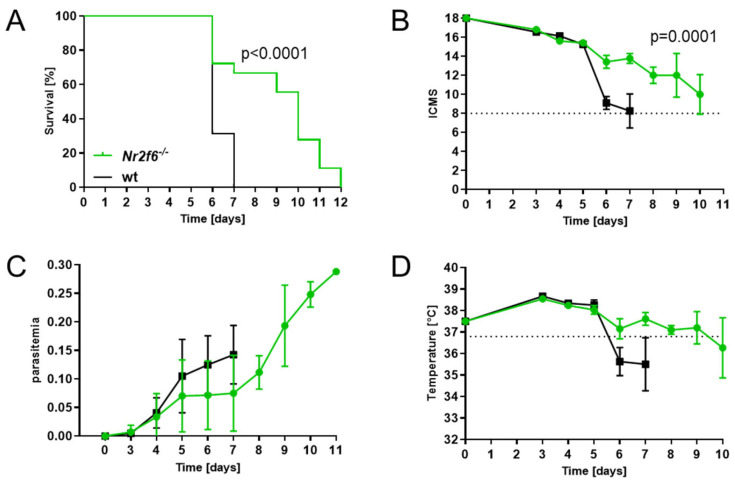
***Nr2f6* deficiency confers partial resistance against cerebral malaria.** (**A**) *Nr2f6*^−/−^ mice show a survival benefit, (**B**) a slower disease progression according to the ICMS without clear clinical signs of ECM, (**C**) a trend to lower parasite load, as well as no severe hypothermia during the ECM vulnerable phase (**D**). n numbers: WT = 16, *Nr2f6^−/−^* = 18. [**B**,**D**] The dashed line represents the cut-off point for terminating the experiment. Log-rank test [**A**], two-way ANOVA [**B**]. Data shown as mean ± SEM.

To determine whether NR2F6 could modulate malaria pathogenesis, we first infected wild-type and whole-body *Nr2f6*-knock-out mice with *Plasmodium berghei* ANKA (*PbA*) parasites (7.5 × 10^4^ parasitized red blood cells, pRBC) [24]. Survival, clinical score, parasitemia, and temperature were monitored daily. *Nr2f6*^−/−^ mice showed a superior survival compared with WT mice, which all had to be sacrificed by day 7 p.i., whereas 70% of *Nr2f6*-knock-out animals survived day 6 (Figure 1A). *PbA*-infected control mice developed CM symptoms from day 6 p.i., and all WT mice succumbed to severe illness by day 7, whereas *Nr2f6*^−/−^ mice developed a moderate score not reaching the cut-off until day 10 p.i. (Figure 1B) Regarding the parasitemia, infected *Nr2f6*-knock-out mice showed a trend to an attenuated phenotype until day 7, but increased dramatically until day 11 p.i., where mice had to be euthanized due to the development of hyperparasitemia (up to 30% parasitemia) (Figure 1C). Comparable to the clinical score and parasitemia, the progression of temperature loss was analogous in *Nr2f6*-knock-out mice, showing less temperature loss early after infection (Figure 1D). This indicates that NR2F6 loss protects mice from ECM but not from malaria-induced hyperparasitemia and death. Taken together, and in strict contrast to *Nr2f6*-knock-out mice, *Nr2f6*-proficient mice exhibited a heightened susceptibility to cerebral malaria pathogenesis.

### 3.1. Delayed Progression of ECM in Nr2f6^−/−^ Mice Correlated with an Intact BBB

**Figure 2:**
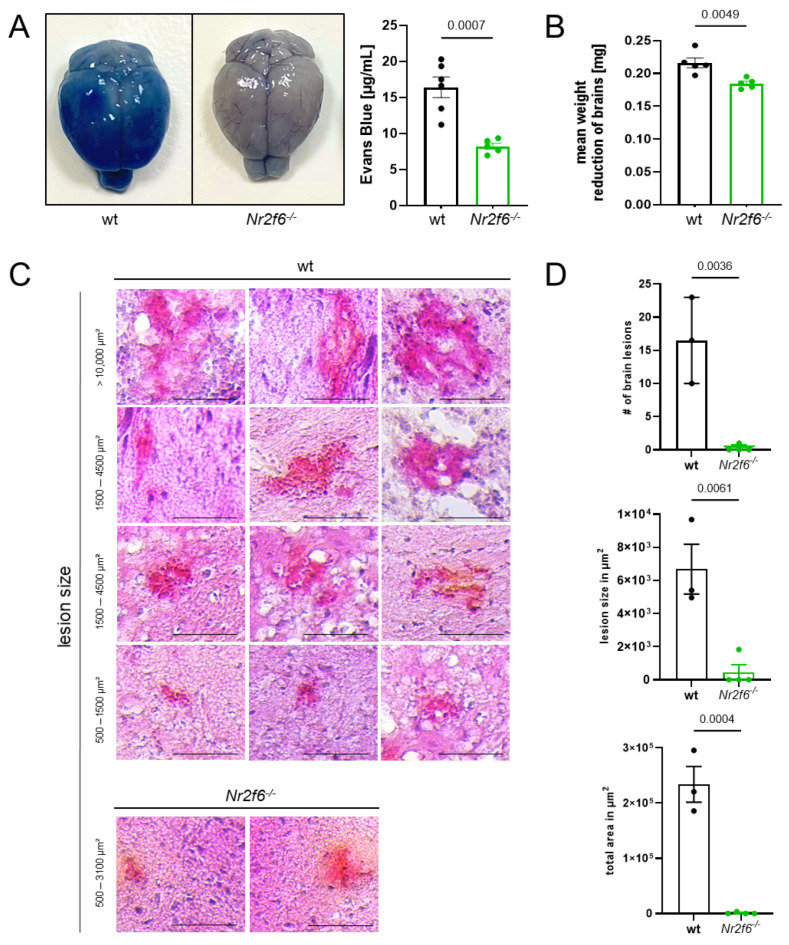
***Nr2f6***^−/−^** mice show a higher BBB integrity and less hemorrhagic lesions.** (**A**) Colorimetric quantification of Evans Blue dye extravasation from isolated brains of *PbA*-infected mice on day 6 p.i. (wt *n* = 6, *Nr2f6*^−/−^ *n* = 5) and (**B**) determination of the amount of volatile liquid in the brain, as a measure of brain swelling, reveals a more intact BBB in *Nr2f6*-knock-out mice (*n* = 5) compared with WT control infected mice (*n* = 5). (**C**) Representative images of H&E-stained brain sections with (**D**) less abundant hemorrhagic lesions, smaller lesions, and therefore account for a markedly smaller total area of lesions in the brains of *Nr2f6*-knock-out animals compared with WT mice infected with *PbA* (day 6 p.i.). Size of scale bars: 200 µm; n numbers: wt = 3, *Nr2f6*^−/−^ = 4. Two-tailed unpaired Student’s *t*-test. Data shown as mean ± SEM.

We next investigated the integrity of the BBB since it is crucial in ECM because its disruption is directly linked to the development of neurological symptoms, brain edema, and death, accompanied by an influx of immune cells into the brain parenchyma [25,26]. Contrary to the expectation that the established hyperactive T cell phenotype would compromise BBB integrity upon *Nr2f6* deficiency [15,18,19,20], knock-out animals exhibited greater BBB stability in the absence of NR2F6 (Figure 2A). In contrast, the brains of infected WT mice showed a permeable BBB, as evidenced by intense staining due to significant extravasation of the i.v.-injected Evans Blue dye (Figure 2A). In line with BBB integrity, we analyzed edema formation using a brain swelling assay. We demonstrated that wild-type mice exhibit increased brain swelling compared with *Nr2f6*-knock-out mice (Figure 2B).

### 3.2. Nr2f6-Deficiency Resulted in the Reduction of Hemorrhagic Brain Lesions

Moreover, when looking at the histological manifestations of *PbA*-infection in *Nr2f6*-knock-out and WT mice, it became apparent that hemorrhagic lesions in the brain, frequently associated with ECM, were less prevalent and pronounced in the absence of *Nr2f6*. In H&E-stained brain sections of *PbA*-infected mice (day 6 p.i.), the size (Figure 2C) and number (Figure 2D) of brain lesions were lower in *Nr2f6*^−/−^ animals than in WT mice. Lesion size varied widely in WT animals from 500 µm^2^ to over 10,000 µm^2^, whereas *Nr2f6*-knock-out mice exhibited fewer lesions, ranging from 500 to 3100 µm^2^. The total area of lesions in the brain was substantially smaller in *Nr2f6*-knock-out mice (Figure 2D). Overall, ECM-associated hemorrhagic tissue damage was less severe in *Nr2f6*-knockout mice.

### 3.3. Nr2f6-Knock-Out Displayed Less Parasite Sequestration and Vessel Obstruction

**Figure 3:**
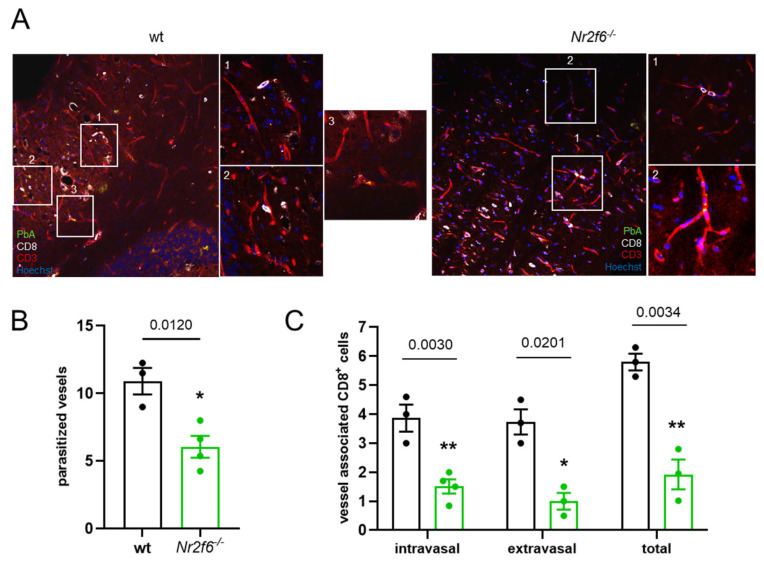
**Less parasites are detectable in *Nr2f6***^−/−^** brains.** (**A**) Representative images of immuno-fluorescent staining of brain sections (20×) of WT (*n* = 3) and *Nr2f6*^−/−^ (*n* = 4) *PbA*-infected mice (day 6 p.i.). Intravasal CD8^+^ T cells (1), extravasal CD8^+^ T cells (2), and parasitized vessel (3) are shown (40×). (**B**) Decreased parasitized vessels manifesting less sequestration of GFP-expressing *PbA* parasites (green) in *Nr2f6*-knock-out mice. (**C**) The amount of intravasal, extravasal, as well as total CD8^+^ T cells (white) per image is lower in association with CD31^+^ ECs (red) of the brain microvasculature (nuclei are illustrated in blue) in *Nr2f6*-knock-out mice than in WT mice. Two-tailed unpaired Student’s *t*-test. Data shown as mean ± SEM. * *p* < 0.05, ** *p* < 0.01.

In line with the improved BBB in *Nr2f6*-knock-out mice infected with *PbA*, we investigated the sequestration of *PbA* parasites to the endothelial cells (ECs) histologically using a GFP-expressing *PbA* (green). This sequestration was more pronounced in wild-type mice than in *Nr2f6*^−/−^ mice (Figure 3A,B). Furthermore, fewer CD8^+^ T cells associated with vessels were found in the ECs of the brain microvasculature (CD31) in *Nr2f6*-knock-out animals (Figure 3A,C). The sequestered lymphocytes were further differentiated based on their location in relation to the brain microvasculature. The invasion of CD8^+^ T cells out of the blood vessels and into the brain tissue (extravasal) was substantially lower in Nr2f6-knock-out than in WT controls. Similarly, the number of intravasal CD8^+^ BILs was approximately 50% lower in Nr2f6^−/−^ than in control mice (Figure 3C). Taken together, the histological analysis of brains from infected Nr2f6-knock-out mice revealed markedly fewer hemorrhages and less vessel obstruction compared with wild-type animals.

### 3.4. Flow Cytometry Analysis Revealed Less Immune Cell Infiltration in Nr2f6-Knock-Out Mice

**Figure 4:**
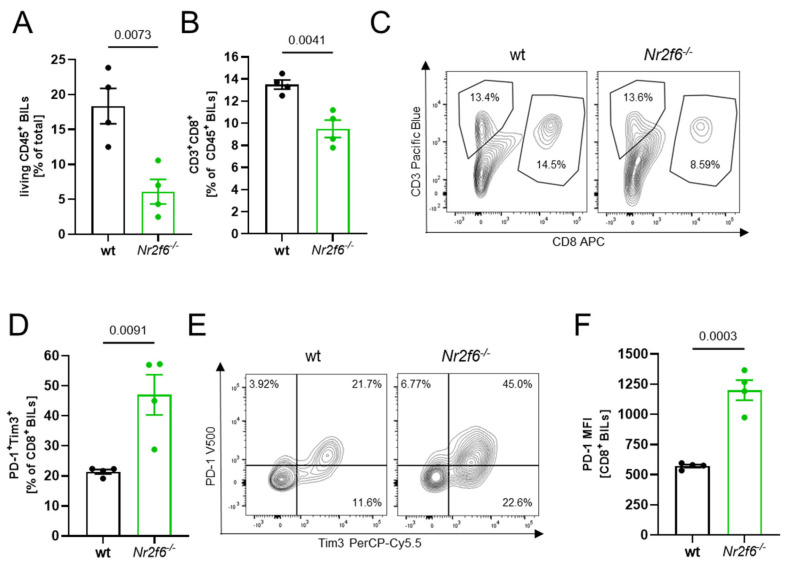
**Lower number of CD8^+^ BILs in *Nr2f6***^−/−^** mice accompanied by higher PD-1 expression.** Flow cytometric analysis of BILs reveals a lower amount of CD45^+^ leukocytes (*p* = 0.0073) of total lymphocytes (**A**) and decreased CD8^+^ T cells (*p* = 0.0041) (**B**,**C**) in the brains of *PbA*-infected *Nr2f6*-knock-out animals compared with WT mice (day 6 p.i.). (**D**,**E**) PD-1^+^Tim3^+^ CD8^+^ BILs were enriched in *Nr2f6*-knock-out mice (*p* = 0.0091). (**E**) Representative FACS blots of PD-1^+^Tim3^+^ T cells. (**F**) CD8^+^ BILs from *Nr2f6*^−/−^ mice showed augmented expression of PD-1 (*p* = 0.0003). Two-tailed unpaired Student’s *t*-test. Data shown as mean ± SEM.

Following the histological analysis revealing fewer CD8^+^ T cells in the brain sections of *Nr2f6*^−/−^ animals vs. WT (Figure 3C—total), we conducted detailed BIL analysis using flow cytometry. In line with the histological analysis and consistent with the reduced number of *PbA*-GFP within the ECM brains in the absence of *Nr2f6*, the flow cytometric analysis of *PbA*-infected (day 6 p.i.) brains revealed a substantial reduction of CD45^+^ leukocytes (Figure 4A) as well as CD8^+^ T cells (Figure 4B,C). A detailed characterization revealed a more “exhausted”, functionally regulated state of CD8^+^ BILs in *Nr2f6*-knock-out infected animals with elevated expression of PD1 and Tim3 (Figure 4D–F). Taken together, the lower number of lymphocytes and the upregulation of exhaustion markers may suppress the cytotoxicity of CD8^+^ T cells, thereby reducing tissue damage to endothelial cells and improving survival in *Nr2f6*-knock-out mice by reducing BBB disruption.

## 4. Discussion

Although scientists have continually explored new ways to combat CM diseases, there is no specific therapy for CM. This has led to an urgent and unmet medical need for research into druggable targets for human CM therapy [4].

ECM models, particularly those using mice infected with *PbA*, have been instrumental in advancing our understanding of the pathogenesis, immunological mechanisms, and potential interventions for CM [27,28]. Notably, several pathological features, including the sequestration of infected erythrocytes, accumulation of leukocytes in brain vessels, and the upregulation of inflammatory mediators, are shared between ECM and human CM, supporting the relevance of these models [26,29]. However, there are important limitations. Animal models cannot replicate all aspects of human disease, and significant species-specific differences exist [30]. For example, while both humans and mice exhibit vascular plugging and neuroinflammation, the precise pattern and extent of leukocyte and parasite sequestration differ. In ECM, brain pathology often develops rapidly and is accompanied by more pronounced neurological symptoms than typically seen in human CM, which displays greater heterogeneity in clinical presentation and outcomes [27,28,29]. The major challenge remains that ECM and human CM only partially overlap on both clinical and pathological grounds. While ECM can recapitulate key pathophysiological elements (such as BBB dysfunction and immune cell interactions), results derived from these models must be interpreted cautiously and validated in human studies when possible [27,28,29]. Thus, ECM models are essential for mechanistic investigation and hypothesis generation, but their translational relevance is contingent upon integration with human data and acknowledgement of their inherent limitations.

ECM infection models in mice have been pivotal in revealing the central role of CD8^+^ T cells in immunopathology. In these models, CD8^+^ T cells infiltrate the brain, interact with endothelial cells, and contribute directly to BBB disruption and neuronal injury through antigen-dependent mechanisms. Depletion or genetic manipulation of CD8^+^ T cells in mice reliably protects against ECM, which underscores their critical contribution to disease onset and severity [31,32,33].

However, the translational relevance of these findings to human CM is an area of ongoing debate. Recent studies confirm that CD8^+^ T cells are present in the brains of patients with human CM, although typically in low and variable numbers [12,13]. While animal models show large accumulations of CD8^+^ T cells as key drivers of pathology, post-mortem analyses of human CM have detected CD8^+^ T cells in contact with cerebral endothelial cells, where they may contribute to BBB disruption and inflammation. The density of CD8^+^ T cells in brain tissue during human CM is generally lower than in experimental models, but their association with blood vessels and meningeal compartments suggests a potential role in human disease progression [12,34].

Disruption of the BBB is one of the hallmarks of CM, so it is crucial to take preventative measures to reduce the severity and fatality of the disease by mitigating or preventing BBB dysfunction. Drugs such as imatinib, FTY720, or Fenozyme have been shown to inhibit vascular leakage, thereby prolonging survival in ECM mouse models, stabilizing the endothelial barrier and preventing BBB breakdown [25,35]. Along these lines, targeting interactions such as LFA-1-mediated leukocyte adhesion or the CD146/Glaectin-9 axis can prevent immune cell accumulation and vascular leakage, thereby protecting BBB integrity [6,25,26]. Therefore, developing a more profound understanding of the role of CD8^+^ cytotoxic lymphocytes in the molecular mechanisms that govern BBB integrity during CM disease is likely to be crucial for the development of promising therapeutic solutions.

In light of the significant limitations in preventing, diagnosing and treating cerebral malaria, our finding that deleting the NR2F6 gene makes mice resistant to ECM by altering T cell migration enhances our understanding of the mechanisms that compromise the BBB integrity during ECM pathology. Our results suggest that inhibiting NR2F6 offers some protection against ECM. Therefore, it appears that NR2F6 suppresses beneficial responses, and its absence creates a brain tissue environment that is less conducive to severe cerebral pathology.

Loss of NR2F6 in ECM is associated with improved survival, reduced BBB integrity loss, fewer cerebral lesions, and fewer CD8 T cells infiltrating the brain in knock-out mice compared with wild-type controls. Mechanistically, NR2F6 is known to regulate immune cell differentiation and function, including myeloid and lymphoid lineages, and to act as a transcriptional suppressor of pro-inflammatory cytokines such as interferon γ. In its absence, immune responses may be modulated in a way that alleviates neuroinflammation and vascular pathology, potentially by reducing the recruitment or activation of pathogenic CD8 T cells in the brain. As BBB disruption and edema are major drivers of ECM pathology, decreased infiltration of CD8 T cells and lower inflammatory signaling can mitigate these effects, contributing to the observed protection in *Nr2f6*-knock-out mice. While the direct link between NR2F6 and immune effector cell trafficking to the brain remains to be fully elucidated, these findings highlight the potential of NR2F6 as a molecular regulator of immunopathology during severe malaria.

As we observed a trend of lower parasitemia in *Nr2f6*-knock-out mice, one could speculate that this results in less pathology, with the observed protection potentially due to enhanced overall resistance to malaria. In this context, regarding systemic immunomodulation and an anti-parasite effect, one could raise the hypothesis that NR2F6 might influence the host response to *Plasmodium* infection because of its high expression in hepatocytes and immune cells such as macrophages [36,37]. There is evidence that changes in the activation state or function of liver-resident macrophages (Kupffer cells) can affect the outcome of malaria. For example, more effective phagocytosis by these cells may help to clear parasites and limit the development of complications [38,39]. However, experimental data directly linking NR2F6 expression to enhanced Kupffer cell phagocytosis and protection from ECM need further investigation. The idea remains plausible, given that modulation of liver immune responses has been shown to critically impact both parasite load and the development of systemic pathology in ECM models [40,41]. As there was only a slight trend in parasitemia with high variation, we speculate that there is no enhanced systemic resistance to the malaria parasite overall. Nevertheless, to fully resolve this issue, it would be necessary to increase the parasitic burden in Nr2f6-knock-out mice compared with controls in order to check for any potentially altered disease progression. In the future, we plan to investigate higher parasite loads in *Nr2f6*-knock-out mice to see if we can still observe improved pathology [42]

In contrast to its role in gut barrier homeostasis, where a lack of NR2F6 expression has been observed to cause detrimental effects on intestinal barrier integrity, promoting colitis and chronic intestinal inflammation [20], loss of NR2F6 expression in ECM appears to prevent BBB dysfunction and reduce immune cell infiltration compared with the wild-type control. This maintains its protective function during ECM pathology.

The role of NR2F6 in ECM differs from its role in autoimmunity. In the latter, NR2F6 deficiency increases pathology due to heightened Th17 responses [16,17], whereas in this *PbA* infectious disease model of ECM, the lack of NR2F6 seems to be advantageous. This highlights the importance of considering the context of the disease when evaluating the function of immune regulators such as NR2F6.

One particularly promising area of study might involve NR2F6 inhibitors [43]. These compounds have attracted attention due to their potential to enhance the body’s immune response to cancer. Remarkably, the newly proposed pathophysiological aspect of BBB dysfunction driven by NR2F6 is providing the first rational insights into encouraging studies with adjunctive therapies that protect against the symptoms of *Plasmodium falciparum* infection in the brain. In the course of testing NR2F6-specific inhibitors, a thorough analysis of side effects, especially those affecting the gut, is required to rule out defects in gut barrier homeostasis.

## 5. Conclusions

This preclinical study has identified NR2F6 as a crucial, non-redundant, and tissue-specific ‘gatekeeper’ within the central nervous system that compromises the BBB, contributing to the severity of ECM. A more intact BBB during NR2F6 deficiency impedes the accumulation of CD8^+^ T cells in the brain, resulting in less damage to the neuronal tissue. Taken together, the evidence from our pioneering research is the first and currently the only preclinical support for the conclusion that inhibiting NR2F6 could be an effective ECM therapy. Therefore, elucidating the mechanism of action of NR2F6 in the pathophysiology of human CM will be helpful when devising future therapeutic treatment strategies.

## Figures and Tables

**Table 1:** Antibody list used for brain and spleen FCM.

Antibodies	Company	Cat.#	Clone	Dilution
CD45 FITC	eBioscience (San Diego, CA, USA)	11-0451-85	30-F11	1:400
CD8 APC	BD Biosciences (Franklin Lakes, NJ, USA)	MA110302	53-6.7	1:400
CD3 Pacific Blue	Biolegend (San Diego, CA, USA)	100214	17A2	1:400
PD-1 BV510	Biolegend	135241	29F.1A12	1:200
Tim3 PerCP-Cy5.5	Biolegend	119718	RMT3-23	1:200
Viability: eFluor 780	BD Biosciences	565388		1:1000

## Data Availability

The raw data supporting the conclusions of this article will be made available by the authors upon request.

## Abbreviations

The following abbreviations are used in this manuscript:

BBB	blood–brain barrier
BIL	brain-infiltrating lymphocyte
CD	cluster of differentiation
CM	cerebral malaria
EC	endothelial cell
ECM	experimental cerebral malaria
FCM	flow cytometry
H&E	Hematoxylin–Eosin
i.v.	intravenous
i.p.	intraperitoneal
ICMS	Innsbruck Cerebral Malaria Score
IF	immunofluorescence
iRBC	infected red blood cells
NR2F6	nuclear receptor subfamily 2, group F, type 6
*PbA*	*Plasmodium berghei ANKA*
*Pf*	*Plasmodium falciparum*
p.i.	post-infection
RT	room temperature
SHIRPA	SmithKline Beecham, Harwell, Imperial College, Royal London Hospital, Phenotype Assessment
WT	wild-type
